# Surgical Treatment of Epiblepharon Effectively Alleviates Keratopathy but Not Astigmatism: A Case-Control Study Utilizing Vector Analysis in East Asian Children

**DOI:** 10.1155/2020/5073895

**Published:** 2020-12-05

**Authors:** Shang-Te Ma, Yao-Lin Liu, Ching-Ju Hsieh, Yo-Shen Chen, Tzu-Hsun Tsai

**Affiliations:** ^1^Department of Ophthalmology, Shuang Ho Hospital, Ministry of Health and Welfare, New Taipei City, Taiwan; ^2^Department of Ophthalmology, National Taiwan University Hospital, College of Medicine National Taiwan University, Taipei, Taiwan; ^3^Department of Ophthalmology, Taipei City Hospital, Taipei, Taiwan; ^4^Department of Surgery, Far Eastern Memorial Hospital, New Taipei City, Taiwan

## Abstract

**Purpose:**

To identify an appropriate surgical indication of epiblepharon by comparing keratopathy and astigmatism outcomes after surgical and medical treatments for epiblepharon in Asian children.

**Methods:**

Children diagnosed with epiblepharon (*n* = 82, age 5.93 ± 2.76 years) with >6 months of follow-up were enrolled. The clinical presentations and cycloplegic refractive status at the baseline and 3 and 6 months after treatment were compared between surgical (91 eyes from 47 children) and nonsurgical (67 eyes from 35 children) groups. The refractive and keratometric astigmatism at each time point were evaluated with vector analysis methods. For Thibos and Horner's method, the astigmatic power vector was decomposed into horizontal and oblique meridians (*J*_0_ and *J*_45_). However, the treatment-induced astigmatism (TIA) vectors were calculated by Alpins' method and depicted by the AstigMATIC software.

**Results:**

In the surgical and nonsurgical groups, the baseline astigmatism magnitude was similar (2.22 ± 1.39 and 2.26 ± 1.46 *D*, *p* = 0.87). The rate of complete resolution of keratopathy at 6 months was 71.4% and 11.5%. The astigmatism magnitude in the surgical group differed among baseline and 3 months (2.25 ± 1.23 *D*) and 6 months postoperatively (1.97 ± 1.28 *D*) (*p* = 0.001). Power vector analyses confirmed a nuanced against-the-rule shift in the surgical group. This trend was especially observed in the subgroup of baseline astigmatism >2.0 *D*. However, the difference in the astigmatism magnitude between surgical and nonsurgical groups, even in highly astigmatic children, was not significant at 6 months.

**Conclusions:**

The improvement of keratopathy in the surgical group was greater than that in the nonsurgical group in consideration of the more advanced severity in the surgery group at baseline. Decreased with-the-rule astigmatism can be observed at 6 months postoperatively, particularly among those with greater baseline astigmatism. However, the amount of change is small, and the outcome does not differ significantly from the nonsurgical treatment. Therefore, surgical indications should majorly base on the severity of symptoms and keratopathy.

## 1. Introduction

Epiblepharon is a congenital eyelid anomaly that is more prevalent in Asian children. This structural lid abnormality usually presents in the lower eyelids and preferentially affects both eyes in about half of the Asian infants [1, 2]. It is characterized by a horizontal and redundant skinfold with underlying pretarsal orbicularis muscle extending over the eyelid margin and thus misdirecting the eyelashes toward the ocular surface [3]. The most common proposed pathophysiology of this condition is a deficiency of attachment of the eyelid retractor fibres to the skin, analogous to the upper lid in Asians with no eyelid crease [4, 5]. The inverted eyelashes may induce ocular surface irritation and corneal erosion; hence, patients may present with nonspecific symptoms, such as frequent eyelid squeezing, blinking, photophobia, tearing, and eye rubbing.

Astigmatism is a type of refractive error in which the eye does not focus light evenly onto the retina through different axes; it may be influenced by a variety of eyelid abnormalities and eyelid positions [6–8]. Epiblepharon is strongly associated with astigmatism, and the proposed underlying mechanisms include mechanical force induced by the redundant skinfold, eyelid squeezing, or aggressive eye rubbing [9–13]. Long-term corneal erosion may also contribute to corneal remodeling and subsequent astigmatism formation [12, 14, 15]. The most common type of astigmatism in epiblepharon patients is the with-the-rule (WTR) type, particularly in those who had long-term persistent corneal erosion [9–12, 14]. Moreover, amblyopia is also more common among children with epiblepharon [10, 11, 16], which may be related to high astigmatism or prolonged keratopathy [14].

Currently, there is no consensus on when and how to manage children with epiblepharon. Children may outgrow epiblepharon; therefore, conservative management, including observation or topical medication, is sometimes suggested [17–19]. Although several surgical techniques have been shown to be effective in relieving keratopathy [2, 20–22], epiblepharon surgery should be offered with caution in consideration of the potential risks, such as exposure to general anaesthesia, wound infection and scarring, lagophthalmos, ectropion, and recurrence [2, 16, 23]. Some authors suggested that surgery may reduce astigmatism by correcting possible underlying mechanisms and recommend early surgery in highly astigmatic epiblepharon patients [14, 24, 25]. However, other studies reported insignificant effects of epiblepharon correction surgery on astigmatism improvement [10–12]. The conflicting results between the studies warrant further investigations.

Part of the discrepancies in previous findings regarding the refractive outcome after epiblepharon surgery may originate from the lack of a proper control group. Furthermore, most studies analyzed the changes in astigmatism depended on the measurement of the absolute value of cylinder power. However, astigmatism comprises parameters of magnitude and axis, and the actual changing effect should be presented as a treatment-induced astigmatism vector. Therefore, the adoption of vector analysis with a proper control group is necessary to analyze astigmatism changes between patients treated with different methods.

The purpose of our study was to compare treatment outcomes, including symptoms, keratopathy, and astigmatism changes after surgical and nonsurgical management of epiblepharon in Asian children. To understand the astigmatic changes more comprehensively, we adopted two methods of power vector analysis, which were conventionally used in cataract and refractive surgery. By clarifying the contradiction in the literature, we hope to determine appropriate management and surgical indications to maximize the effectiveness of treatments for epiblepharon in children.

## 2. Materials and Methods

**Figure 1:**
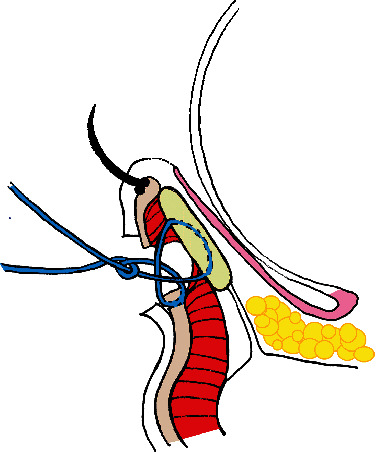
Illustration of rotating suture technique. Averagely 3 interrupted tarsus eversion sutures with absorbable 6-0 Vicryl was performed. Cosmetic tape was covered on the surgical wound to provide protection and enhance wound healing until postoperative 1 month.

Children with epiblepharon who were younger than 18 years and were treated and followed up by a single surgeon (THT) for more than 6 months at the National Taiwan University Hospital from 2007 to 2016 were enrolled. The study protocol was approved by the Research Ethics Committee of the National Taiwan University Hospital. The severity of keratopathy related to epiblepharon with cilia touch was assessed based on the grading system proposed by Kim et al. [20]. The grading of keratopathy severity was divided into grade 0 to grade 3 according to the fluorescent staining pattern under biomicroscopy with cobalt blue light. Grade 0 keratopathy indicated that no punctate or confluent fluorescent staining was seen. The fluorescent staining restricted to the peripheral medial half of the cornea was grade 1, while staining involving peripheral temporal half of the cornea was grade 2. Once the corneal center within the pupillary margin was involved, it was referred to grade 3 keratopathy. Surgical indications included persistent corneal erosion involving the pupil area under biomicroscopy with fluorescein staining (i.e., grade 3) or refractory subjective symptoms, such as irritation, tearing, frequent eye rubbing, or eye squeezing reported by caregivers. Informed consent of the surgery was explained and signed by the parents/guardians before the operation. Patients with combined amblyopia and definite systemic or ocular anomalies, including those with Down syndrome, congenital cataract, lid coloboma, or other congenital anomaly, were excluded from the study. Surgery was performed under general anaesthesia. The surgeon marked the excessive skin in a fusiform shape at the nasal quadrant of the lower lid. Skin incision and removal were done with a number 15 blade. Only a small portion of the orbicularis muscle was excised, and the remaining were dissected with small scissors to expose the tarsus. Tarsal eversion was performed with three interrupted 6-0 Vicryl sutures, which also apposed the anterior lamella of the incision wound and the underlying tarsus (Figure 1). Finally, the skin wound was closed with interrupted 8-0 Vicryl sutures and covered with cosmetic tape. Patients with epiblepharon who did not receive surgery were treated with topical lubricative agents (Artelac® 3.2 mg/mL Eye Drops; Dr Gerhard Mann chem-pharm Fabrik GmbH, Berlin, Germany; or TEARS NATURALE® Eye Drops; Alcon, Puurs, Belgium) two to four times a day. The treatment responses of surgery or medical treatment were repeatedly evaluated in a 3-month interval.

Data on sex, age of treatment, refractive status, astigmatism type, and grading of keratopathy were collected from medical records. Status at the baseline and at 3 and 6 months after treatment was compared between the surgical and nonsurgical groups. Cycloplegic refraction was obtained with topical instillation of Mydrin-P (Santen, Osaka, Japan) 3 times, at 5-minute intervals, and autorefraction (Topcon KR 8000PA; Topcon Corp., Tokyo, Japan) was assessed at 15 minutes after the last drop of Mydrin-P solution was applied. Data of keratometric astigmatism were also obtained from keratometry using the same machine. Changes in both refractive and keratometric astigmatism between before and after treatment was measured with the vector analysis methods advocated by Thibos and Horner [26] and Alpins [27]. The astigmatic axis of the left eye was mirrored (e.g., 165° to 15°) so that the right eye and left eye data could be integrated for vector analysis. In Thibos and Horner's method, the cylinder power (C) and axis (*α*) are divided into two vectors: *J*_0_ = (−C/2)cos(2*α*) and *J*_45_ = (−C/2)sin(2*α*), representing the astigmatism power at axis *α* = 0° = 180° and *α* = 45°, respectively. In terms of Alpins' method, we calculated the treatment-induced astigmatism (TIA) vector at 3 and 6 months after treatment. Here, TIA corresponds to surgically induced astigmatism (SIA) in the standard terminology of Alpins' method, and we use this term to represent the astigmatism change after the treatment (surgery or conservative treatment) in our study. The standard single-angle polar plots of TIA were drawn using the AstigMATIC software [28]. Subgroup analysis was performed to determine the effect of surgery on astigmatism change in cases with different baseline levels of astigmatism.

The results were analyzed with Excel (Microsoft, Inc. Redmond, Washington, USA), SPSS 26.0 (IBM Corp., Armonk, NY, USA), and *R* version 4.0.0 (R Foundation for Statistical Computing, Vienna, Austria). Demographic data, grading of keratopathy, and refractive status were compared between the surgical and nonsurgical groups by using *t*-tests for continuous variables and Fisher's exact test or the chi-square test for categorical variables. Refractive data were collected from both eyes of most children. Therefore, for each refractive parameter (i.e., spherical equivalent, magnitude of astigmatism, *J*_0_, and *J*_45_), repeated-measure analysis of variance (ANOVA) regarding the laterality of eye as a within-subject variable was used to evaluate the within-group differences among the pre-treatment, and 3- and 6-month post-treatment time points, and also the between-group differences at each time point. A multivariable linear mixed-effect model was used to explore the effect of surgery on astigmatic change and its interaction with age and baseline astigmatism. *p* < 0.05 was defined as statistically significant.

## 3. Results

In total, 158 eyes of 82 children were enrolled, including 91 eyes from 47 patients in the surgical group and 67 eyes from 35 patients in the nonsurgical group. Most patients had bilateral involvement (76/82, 92.7%). The baseline demographic data are shown in Table 1. The subjective ocular irritation symptoms and the severity of keratopathy were more prominent in children in the surgical group. They reported symptoms, including frequent eye rubbing (51.1%), irritation (38.3%), photophobia (23.4%), epiphora or red eye (21.2%), and frequent eye squeezing (12.8%). There was no significant difference between the two groups in terms of age at treatment, sex, spherical equivalent, magnitude of astigmatism, *J*_0_ and *J*_45_ in Thibos and Horner's astigmatic vector analysis, and the type of astigmatism. The major astigmatism type in both groups was WTR astigmatism.

The baseline level of keratopathy in both surgical and nonsurgical groups showed significant inter-eye correlation within each patient. The Spearman correlation coefficients were 0.717 (*p* < .001) and 0.682 (*p* < .001) in the surgical and nonsurgical group, respectively. Therefore, for patients with uneven severity in both eyes, the eye with higher grading was collected. If both eyes had equal severity, only right eye was collected. The results are shown in Table 2. The baseline distribution of the grading of keratopathy in both groups showed significant difference (*p* < .001). In the surgical group, 57.4% (27 of 42) of patients had grade 3 keratopathy, while only 8.6% (3 of 35) of patients reached such severity in the nonsurgical group. After surgical correction, the severity of keratopathy improved significantly, with 71.4% (30 of 42) of the patients free of keratopathy at 6 months postoperatively. The resolution or improvement of subjective symptoms was reported by all patients in the surgical group. There was no recurrent case during the 6-month postoperative period. In the nonsurgical group, keratopathy showed borderline improvement (*p* = 0.077). Only 11.5% (3 of 26) of patients had complete resolution of keratopathy in the nonsurgical group. Comparing the keratopathy grading between both groups at post-treatment 6-month period, the surgical group showed a significant better outcome (*p* < .001). Overall, the more severe baseline keratopathy in the surgical group greatly improved after the treatment and reached an even better level than that in the nonsurgical group.

The refractive status at different treatment stages in both groups is shown in Table 3. A progressive myopic shift was observed in both the surgical and nonsurgical groups (*p* = 0.04 and <0.0001, respectively), but no significant difference was noted between the two groups at 3 and 6 months after treatment (*p* = 0.54 and 0.46, respectively). In the surgical group, the magnitude of astigmatism differed significantly within groups at baseline and 3 and 6 months after treatment (*p* = 0.019), which decreased from 2.22 *D* at baseline to 1.97 *D* at 6 months post-treatment. In contrast, the magnitude of astigmatism in the nonsurgical group remained stable throughout the follow-up period (*p* = 0.87). However, when we compared the surgical and nonsurgical groups, no significant between-group differences in the magnitude of astigmatism were noted at 3 and 6 months postoperatively (*p* = 0.98 and 0.45, respectively). For *J*_0_ and *J*_45_ of astigmatism as determined by Thibos and Horner's power vector analysis method, no significant within-group difference was noted in the surgical or nonsurgical group (*J*_0_: *p* = 0.09 and 0.80; *J*_45_: *p* = 0.86 and 0.05, respectively); there were also no between-group differences at 3 and 6 months post-treatment (*J*_0_: *p* = 0.88 and 0.73, *J*_45_: *p* = 0.29 and 0.50, respectively).

**Figure 2:**
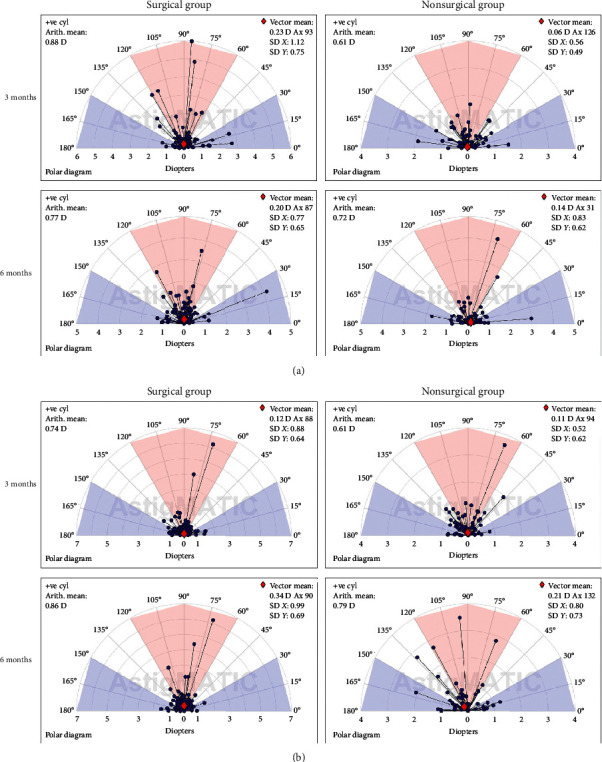
Single-angle polar plots of treatment-induced astigmatism (TIA) vector, including refractive (a) and keratometric (b) astigmatism at postoperative 3 and 6 months using Alpins' method.

We analyzed the refractive and keratometric astigmatism change using both Thibos and Horner's and Alpins' methods. The comparative results of the *J*_0_ and *J*_45_ changes by Thibos and Horner's method at 3 and 6 months post-treatment in both groups are shown in Table 4. At 3 months, no significant differences in changes in *J*_0_ and *J*_45_ were noted between the two groups. At 6 months, a significant decreasing change in *J*_0_ in both the refractive and keratometric measurement in the surgical group was noted, as compared with that in the nonsurgical group (refractive: −0.10 *D* vs. 0.03 *D*, *p* = 0.03; keratometric: −0.17 *D* vs. −0.01 *D*, *p* = 0.03). This suggests more against-the-rule (ATR) change in the surgical group. No significant difference was noted in the change in *J*_45_ at 6 months. Using Alpins' method, we calculated the TIA vector, including refractive and keratometric astigmatism of each eye in the surgical and nonsurgical groups. The standard single-angle polar plots are shown in Figure 2. In the surgical group, the vector means obtained from the refractive and keratometric values at 3 and 6 months ranged from 0.12 to 0.34 *D* in magnitude and were all in a consistent 90-degree direction, indicating an ATR change. This consistency was not observed in the nonsurgical group. Overall, the results of vector analysis from both Thibos and Horner's and Alpins' methods consistently showed a small ATR astigmatic change in the surgical group, which was not observed in the nonsurgical group.

**Figure 3:**
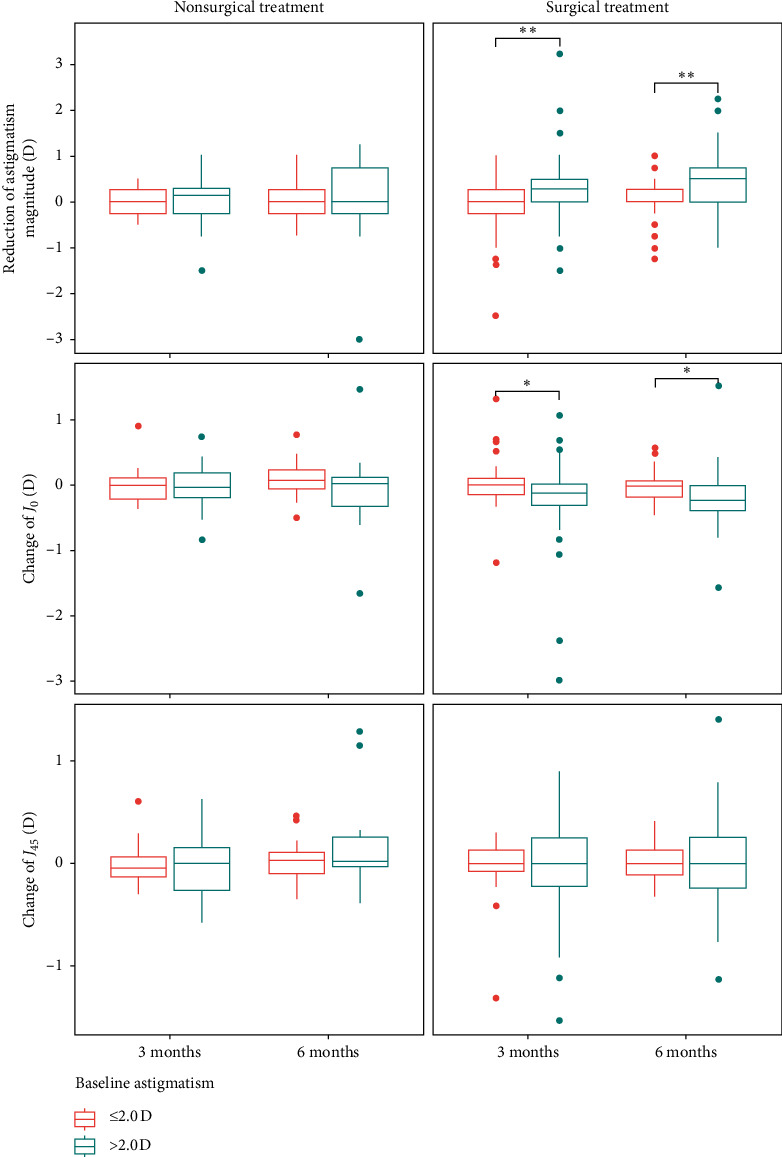
Change of astigmatism magnitude, *J*_*0*_ and *J*_*45*_ of the two study groups at postoperative 3 and 6 months, stratified by baseline astigmatism magnitude (≤2.0 and >2.0 diopters). ^*∗*^*p* value < 0.05; ^*∗∗*^*p* value < 0.01.

We further divided our study population by baseline astigmatism magnitude into two subgroups (≤2.0 *D* and >2.0 *D*, Figure 3) for a subgroup analysis. In the surgical group, eyes with baseline astigmatism >2.0 *D* showed a significant reduction in astigmatism magnitude (0.34 ± 0.78 *D* and 0.50 ± 0.72 *D* at 3 and 6 months, respectively), compared with eyes with baseline astigmatism ≤ 2.0 *D* (0.04 ± 0.56 *D* and 0.08 ± 0.87 *D* at 3 and 6 months, respectively). Eyes with baseline astigmatism >2.0 *D* also showed significant change in *J*_*0*_ (−0.23 ± 0.67 *D* and −0.20 ± 0.47 *D* at 3 and 6 months, respectively), compared with eyes with baseline astigmatism ≤ 2.0 *D* (−0.01 ± 0.33 *D* and −0.06 ± 0.56 *D* at 3 and 6 months, respectively). The finding suggested a more decrease of WTR astigmatism in eyes with high baseline astigmatism after the surgery. However, when we compared the refractive astigmatism status, not the change, among the subgroups, neither the within-group differences among different time points nor the between-group differences at each time point reached the significance level (Table 5).

Finally, potential factors influencing the change in the astigmatism magnitude after the treatment were explored using a multivariable linear mixed-effect model (fixed effect: age [<6 years or ≥6 years], treatment [surgery or nonsurgery), baseline astigmatism amplitude [≤2.0 *D* or >2.0 D), and two interaction terms [treatment and age, treatment and baseline astigmatism]; and random effect: eye [right or left eye], time [3 months or 6 months]). The results showed that only the interaction term of surgery and baseline astigmatism had a significant positive effect on the reduction of astigmatism magnitude (*p* = 0.01). Age had no significant effect. Among children who received surgical correction, the least-square mean of astigmatism reduction derived from the model was 0.44 *D* and 0.02 *D* in those whose baseline astigmatism was >2.0 *D* and ≤2.0 *D*, respectively.

## 4. Discussion

Our study found that in Asian children with epiblepharon, surgical treatment alleviated clinical symptoms and keratopathy. Small-amplitude ATR shift, or decreased WTR astigmatism, can be observed at 6 months postoperatively, particularly in those with baseline astigmatism exceeding 2.0 *D*. However, the change was small and thus did not reach statistical significance when compared with children who received conservative treatment.

In our study, the keratopathy resolved significantly after surgical correction of epiblepharon, with 71.4% of patients in the surgical group were free of keratopathy. Conservative management with topical lubricants had limited effects on keratopathy improvement. Only 11.5% of patients in the nonsurgical group had complete resolution of keratopathy during the follow-up period. The improvement of keratopathy in the surgical group was greater than that in the nonsurgical group in consideration of the more advanced severity in the surgery group at baseline; however, it might be too robust to draw a conclusion that surgery is superior to nonsurgical treatment in the setting of the retrospective design of our study. Conservative treatments such as topical lubricative agents might still be effective in certain patients. Moreover, although no recurrence of epiblepharon or keratopathy was noted at 6 months postoperatively in our study, Kim et al. [16] reported a 3.6% recurrence and a 1.9% reoperation rate in their 9-year study. A long-term follow-up is necessary to provide a more accurate assessment of recurrence.

Previous studies that investigated the effect of epiblepharon correction on astigmatism changes in Asian children showed conflicting results. Shih and Huang [12] investigated school-age children with epiblepharon in southern Taiwan and proposed that astigmatism was correlated with preoperative keratopathy severity and the magnitude of astigmatism did not change significantly after blepharoplasty. Yang et al. [10] found that operation improved the best corrected visual acuity in amblyopic patients but did not significantly change the magnitude of astigmatism after 1 year of follow-up. Preechawai et al. [11] performed a 2-year follow-up in Singapore and also reported that astigmatism did not improve significantly after operation in different age subgroups. Nevertheless, later studies performed subgroup analysis according to the severity of astigmatism and age and revealed that astigmatism was improved significantly in children with high astigmatism [14, 25], who were under six years [14] or around school age [24, 25]. The disparities between studies may originate from the baseline characteristics of the recruited children, including their age and ethnicity, the surgical methods employed in the studies, the follow-up period, the analytic methods of astigmatic change, as well as the inclusion and the definition of a control group in the study design.

In our study, the most common type of astigmatism in epiblepharon children was the WTR type (around 80%), which was compatible with the findings of previous reports (60.7–90.5%) [9–12, 14, 25]. We observed that the magnitude of both refractive and keratometric astigmatism reduced significantly in the surgical group at 6 months postoperatively, particularly in children with higher baseline astigmatism. Further astigmatic vector analysis using both Thibos and Horner's and Alpins' methods confirmed a small ATR astigmatic change in the surgical group by 6 months after treatment. When comparing between the surgical and nonsurgical groups, no significant between-group difference was noted at 3 or 6 months postoperatively in terms of the magnitude of astigmatism and *J*_0_ and *J*_45_ of astigmatism, even in the sub-group of high astigmatism (>2D) children. The overall findings suggested that surgery did have any effect on alleviating WTR astigmatism; however, it might not be clinically significant when comparing the outcome with conservative treatment.

Some studies have found that the reduction of astigmatism after surgery was more significant at younger age (around or less than 6 years old) [14, 25]. Our multivariable linear mixed-effect model revealed that the age of treatment (younger than 6 years old or not) did not have a significant positive effect in reducing astigmatism magnitude, i.e., age might not be a major concern for epiblepharon surgery when considering astigmatism improvement according to the findings of our study. However, surgery is indicated in young children with clinical symptoms and severe keratopathy, which may lead to amblyopia formation [23].

The proposed mechanisms of astigmatism in epiblepharon children included mechanical force induced by the skinfold, eyelid squeezing or aggressive eye rubbing, and long-term corneal erosion with corneal remodeling [9–15]. However, the observations of children in our study (around 6 years old) did not support these mechanisms. In the present study, the symptoms and keratopathy in the surgical group were significantly more severe than the nonsurgical group, but the baseline astigmatism magnitude was similar between them. Besides, the surgical correction effectively alleviated symptoms and keratopathy but only had minimal effect on astigmatism reduction. We speculate that the contributing factor for the astigmatism formation in epiblepharon act at a much earlier age and lead to an irreversible corneal astigmatic change. Further longitudinal investigations in young epiblepharon children from age 1 to 5 are required.

Our study was limited by its retrospective nature, possible allocation bias for the treatment group, and a short follow-up duration. For instance, the level of keratopathy was significantly different between surgical and nonsurgical groups at enrollment, which precluded an equitable comparison of different treatments. A long-term, prospective, randomized study is needed for a better comparison of the outcomes of epiblepharon management in Asian children.

## 5. Conclusion

Surgical correction of epiblepharon improved keratopathy and the subjective symptoms related to ocular surface irritation. Children with baseline astigmatism more than 2.0 *D* may further benefit from surgery with improvement in astigmatism at 6 months after surgery, although the effect is small. We suggest that the indication for surgery in children with epiblepharon should be based on the severity of their clinical symptoms and keratopathy.

## Figures and Tables

**Table 1:** Demographic data, symptoms, severity of keratopathy, and refractive status of the study population.

	Children with epiblepharon		*p* value
	Surgical treatment (91 eyes of 47 patients)	Non-surgical treatment (67 eyes of 35 patients)	
	Mean ± SD, *n* (%)		
Age (year)	5.93 ± 2.76	5.87 ± 2.10	0.87
Sex (female)	28 (59.6)	16 (45.7)	0.75
Severity of keratopathy (eyes)			
Grade 0	0 (0)	0 (0)	<0.001^∗^
Grade 1	4 (4.4)	58 (86.6)	
Grade 2	35 (38.5)	6 (9.0)	
Grade 3	52 (57.1)	3 (4.4)	
Spherical equivalent (D)	−0.58 ± 2.88	−0.70 ± 2.90	0.78

Astigmatism			
Magnitude (D)	2.22 ± 1.39	2.26 ± 1.46	0.87
Vector *J*_0_ (D)	0.97 ± 0.80	0.94 ± 0.86	0.83
*J*_45_ (D)	0.05 ± 0.35	−0.05 ± 0.44	0.14

Type			
With-the-rule	73 (80.2)	55 (82.0)	0.28
Against-the-rule	4 (4.4)	6 (9.0)	
Oblique	14 (15.4)	6 (9.0)	

SD, standard deviation; SE, spherical equivalent; D, diopter. Magnitude of astigmatism indicate the absolute value of cylinder power regardless of the axis. Thibos and Horner's vector analysis method was used to represent the vector of astigmatism. ^*∗*^*p* < 0.05.

**Table 2:** Severity of keratopathy in the two groups at baseline and 6 months after treatment.

	Keratopathy grading	After treatment	6 months after treatment	*p* value
		Number of children (%)		
Surgical treatment	Grade 0	0 (0)	30 (71.4)	<0.001^*∗*^
	Grade 1	2 (4.3)	10 (23.8)	
	Grade 2	18 (38.3)	2 (4.8)	
	Grade 3	27 (57.4)	0 (0)	
	Total	47	42	

Nonsurgical treatment	Grade 0	0 (0)	3 (11.5)	0.077
	Grade 1	27 (77.1)	21 (80.7)	
	Grade 2	5 (14.2)	2 (7.7)	
	Grade 3	3 (8.6)	0 (0)	
	Total	35	26	

*p* value		<0.001^*∗*^	<0.001^*∗*^	

Keratopathy grading data were collected from one eye of each child for analysis. For patients with uneven severity in both eyes, the eye with higher grading was collected. If both eyes had equal severity, only right eye was collected.*p* values were calculated by Fisher's exact test. ^*∗*^*p* < 0.05.

**Table 3:** Refractive status of the two study groups at baseline and 3 and 6 months after treatment.

	Surgical treatment					Nonsurgical treatment				
	Number of eyes	Spherical equivalent	Astigmatism			Number of eyes	Spherical equivalent	Astigmatism		
			Magnitude	Power vector				Magnitude	Power vector	
				J_0_	J_45_				J_0_	J_45_
	Mean diopters ± SD					Mean diopters ± SD				
Before treatment	91	−0.58 ± 2.88	2.22 ± 1.39	0.97 ± 0.80	0.05 ± 0.35	67	−0.70 ± 2.90	2.26 ± 1.46	0.94 ± 0.86	−0.05 ± 0.44

3 months after treatment	83	−0.75 ± 3.00	2.25 ± 1.23	0.95 ± 0.73	0.04 ± 0.46	61	−1.11 ± 2.78	2.22 ± 1.48	0.96 ± 0.82	−0.06 ± 0.44

6 months after treatment	82	−0.85 ± 2.97	1.97 ± 1.28	0.88 ± 0.68	0.02 ± 0.38	51	−1.27 ± 3.20	2.17 ± 1.60	0.92 ± 0.89	−0.04 ± 0.42

*p* value		0.04^*∗*^	0.019^*∗*^	0.09	0.86		<0.0001^*∗*^	0.87	0.80	0.05

SD, standard deviation. The magnitude of astigmatism indicates the absolute value of cylinder power, regardless of the axis. Thibos and Horner's method was used in the power vector analysis of astigmatism. *p* values were calculated using the repeated-measure ANOVA, regarding the laterality of eye as a within-subject variable. ^*∗*^*p* < 0.05.

**Table 4:** Refractive and keratometric change of *J*_0_ and *J*_45_ from Thibos and Horner's power vector analysis at 3 and 6 months after treatment.

	Children with epiblepharon		
	Surgical treatment	Nonsurgical treatment	*p* value
3 months after treatment			
Number of eyes	83	61	

Refractive			
J_0_ change	−0.12 ± 0.56	−0.01 ± 0.28	0.07
J_45_ change	−0.01 ± 0.38	−0.03 ± 0.25	0.39

Keratometric			
J_0_ change	−0.06 ± 0.44	−0.05 ± 0.44	0.45
J_45_ change	0.004 ± 0.32	−0.01 ± 0.31	0.41

6 months after treatment			
Number of eyes	82	51	

Refractive			
J_0_ change	−0.10 ± 0.39	0.03 ± 0.42	0.03^*∗*^
J_45_ change	0.01 ± 0.32	0.06 ± 0.31	0.25

Keratometric			
J_0_ change	−0.17 ± 0.50	−0.01 ± 0.40	0.03^*∗*^
J_45_ change	0.002 ± 0.34	−0.10 ± 0.37	0.05

SD, standard deviation. *p* values were calculated using Student's *t*-test. ^*∗*^*p* < 0.05.

**Table 5:** Refractive astigmatism status of the two study groups at baseline and 3  and 6 months after treatment, stratified by baseline astigmatism magnitude (≤2.0 and >2.0 diopters).

Baseline magnitude	Surgical treatment				Nonsurgical treatment			
	≤2.0 *D*		>2.0 *D*		≤2.0 *D*		>2.0 *D*	
Pre-treatment								
Number of eyes	47		44		37		30	
Magnitude (D)	1.13 ± 0.63		3.39 ± 0.96		1.26 ± 0.54		3.50 ± 1.28	
*J*_0_/*J*_45_ (D)	0.46 ± 0.39	0.07 ± 0.23	1.52 ± 0.78	0.06 ± 0.45	0.43 ± 0.44	−0.04 ± 0.30	1.57 ± 0.83	−0.05 ± 0.57
3 months after treatment								
Number of eyes	39		44		33		28	
Magnitude (D)	1.34 ± 0.78		3.06 ± 0.96		1.23 ± 0.53		3.39 ± 1.37	
*J*_0_/*J*_45_ (D)	0.57 ± 0.44	0.08 ± 0.29	1.28 ± 0.77	0.01 ± 0.57	0.48 ± 0.35	−0.06 ± 0.30	1.52 ± 0.86	−0.05 ± 0.56
6 months after treatment								
Number of eyes	41		41		29		22	
Magnitude (D)	1.05 ± 0.77		2.89 ± 1.00		1.17 ± 0.63		3.47 ± 1.56	
*J*_0_/*J*_45_ (D)	0.45 ± 0.41	0.05 ± 0.22	1.31 ± 0.62	−0.003 ± 0.49	0.47 ± 0.40	−0.05 ± 0.26	1.53 ± 1.00	−0.02 ± 0.58
*p* value	0.86		0.14		0.86		0.67	
	0.76	0.63	0.20	0.85	0.50	0.87	0.81	0.06

SD, standard deviation, *D*, diopter. Magnitude of astigmatism indicates the absolute value of cylinder power regardless of the axis. Thibos and Horner's method was used in the power vector analysis (*J*_0_/*J*_45_) of astigmatism. *p* values were calculated using the repeated-measure ANOVA, regarding the laterality of eye as a within-subject variable. ^*∗*^*p* < 0.05.

## Data Availability

The Data are available upon request to the corresponding author.
